# Satellite Launcher Navigation with One Versus Three IMUs: Sensor Positioning and Data Fusion Model Analysis

**DOI:** 10.3390/s18061872

**Published:** 2018-06-07

**Authors:** Yanick Beaudoin, André Desbiens, Eric Gagnon, René Landry

**Affiliations:** 1 Département de Génie Électrique et de Génie Informatique, Université Laval, 1065 Avenue de la Médecine, Québec, QC G1V 0A6, Canada; Andre.Desbiens@gel.ulaval.ca; 2 Defence Research and Development Canada, 2459 Route de la Bravoure, Québec, QC G3J 1X5, Canada; Eric.Gagnon@drdc-rddc.gc.ca; 3 Département de Génie Électrique, École de Technologie Supérieure, 1100 Rue Notre-Dame Ouest, Montréal, QC H3C 1K3, Canada; renejr.landry@etsmtl.ca

**Keywords:** INS, GPS, attitude reference sensor, navigation, data fusion, sensors positioning

## Abstract

Using multiple IMUs allows both their distribution along vehicle structures and a reliance on integration methods, which is not possible with a single IMU. This paper addresses the issue of relying on three IMUs instead of only one of a higher quality in the context of a satellite launcher. The impact of the IMU positions was tested by comparing collocated IMUs against IMUs installed in the head of each launcher stage. For multi-IMU configurations, three integration methods were tested: all IMUs fused in a single INS, multiple INSs fused in a stacked filter, and multiple INSs fused in a stacked filter with geometrical constraints. All navigation solutions were aided by a three-axis attitude reference sensor and were tested with and without a GPS receiver. The results show that distributing IMUs along the launcher structure does not improve navigation performances compared to having them collocated. The fusion of multiple IMUs in one INS provides equivalent results as one IMU. However, fusing multiple INSs greatly reduces estimation errors. Performances are further improved with the addition of geometrical constraints. During long GPS outages, relative velocity and position constraints should not be exploited, as they may lead to filter divergence.

## 1. Introduction

For years now, inertial navigation has been successfully exploited in space vehicles [1]. However, achieving adequate performances requires high quality units. In small launchers targeting low orbits, the cost of the navigation solution may represent a significant proportion of the total cost [2]. The development of MEMS (Micro Electro Mechanical Systems) technologies leads to a reduction of the cost and size of IMUs. Although MEMS sensors provide lower performances than high-end units, their small size and low cost allow them to be integrated into redundant configurations [3,4]. Relying on multiple IMUs is frequent in space vehicles [5,6,7]. However, to the authors’ knowledge, these IMUs are used in independent navigation solutions in order to detect and isolate inertial sensor failures or as a safeguard navigation solution. Here, the aim is to evaluate the impact on the navigation performances of fusing all the inertial sensors in one navigation solution.

Multiple IMUs can be fused together or individual inertial sensors may be used. Sensors may be installed in an orthogonal or non-orthogonal configuration. The latter configuration constitutes what is known as a skew-redundant IMU [8,9,10]. Inertial sensors can be arranged such that their sensitive axes are in opposite directions in order to cancel systematic errors which are correlated among sensors [11]. An array of inertial sensors can be integrated in one package [12,13] or sensors may be distributed along the vehicle [8,14]. Many researches have investigated the optimal geometry required to fuse an inertial sensor cluster [8,9,15,16,17,18,19,20]. The position and orientation of inertial sensors can be selected to maximize the volume of information on one axis—for example, to compensate for the GPS vertical error [10]—or according to the vehicle dynamics [21]. When triads of orthogonal accelerometers and gyroscopes are involved, the volume of information provided is unaffected by the orientation of IMUs [18]. However, skewed IMUs provide better fault detection and isolation performances [22,23]. To simplify the analysis, considering that this work focuses on the navigation precision, IMUs with three gyroscopes and accelerometers orthogonally configured are used.

While IMUs often include gyroscopes and accelerometers, it is possible to have IMUs with accelerometers only, also known as gyro-free IMUs [19,24,25,26,27,28]. If the accelerometers are positioned properly, the axial acceleration can be isolated from the angular acceleration. However, most successful implementations of gyro-free IMU are in applications with high angular rates [29] as the angular dynamics information provided by the accelerometer is proportional to the square of the angular velocity [30]. On the other side, the angular rate sensed by the accelerometers is proportional to the square of the distance between the accelerometers [19,30,31]. Considering the size of a satellite launcher, long lever arms are possible. Furthermore, the principle of distributing the accelerometers can be combined with gyroscopes in order to exploit both sensor types to measure the angular dynamics of the vehicle [30,31]. On a satellite launcher, where the trajectory is mostly aimed in one direction, the propagation of the attitude estimation error into the velocity and position estimates is mostly due to the yaw and pitch estimation errors. To better exploit accelerometer measurements and thereby reduce the yaw and pitch estimation errors, the IMUs should be distributed along the launcher axis [21].

Many approaches can be adopted to fuse multiple IMUs [8,9]. Among them, all IMU data can be fused together to create a synthetic IMU [32,33], which is also referred to as a virtual IMU [34]. The virtual IMU data is then sent to a single INS. To that end, all sensor measurements must be represented in the virtual IMU frame and position. When considering a rigid structure, the gyroscope measurements only need to be rotated in the appropriate reference frame. However, the accelerometer measurements are also affected by the lever arm between the sensors and the virtual IMU reference position, which is similar to the size-effect presented in Savage [35]. Therefore, a translation of the acceleration from each sensor position to its equivalent in the virtual IMU position [36,37,38], also known as the Grubin transformation [36], is performed. Even though it can provide accuracy improvements, in many navigation solutions, the lever arm is considered negligible, and the Grubin transformation is omitted [39]. The simplest method to create a virtual IMU is to average the specific force and angular velocity measurements of all IMUs [11,39]. Another method involves fusing all IMU data using a least squares or a Kalman filter. This approach provides better performances since individual sensor biases can be estimated within the data fusion of the virtual IMU [39,40]. However, in this method, observability problems were noted [10,22], which prevent the correct estimation of biases. Bancroft and Lachapelle [39] confirmed that, when the angular acceleration, angular velocity, and specific force are estimated with only two IMUs, using least squares, a maximum of 8 states out of 9 are observable. One advantage of the virtual IMU is that it can be integrated as a single IMU within an existing navigation solution and hence prevents software modification [22,32]. However, this approach does not allow one to exploit the aiding sensor measurements to estimate the individual inertial sensor errors, which makes this solution sub-optimal [22,33].

Several solutions can be used to overcome the limitation of the virtual IMU. For example, with the extended mechanization, the standard strapdown navigation equations are modified to use redundant measurements as well as to estimate all sensor errors [10,22,32,33]. This solution may be implemented in one or multiple navigation processors [33], and it can be combined with the virtual IMU where the estimated errors are removed from the raw sensor measurements [32]. In a previous work, the authors have presented a navigation solution aided by a stochastic model of the vehicle dynamics [41]. This solution approximates the angular velocity and acceleration as random walks with known driving noise variances based on the dynamics of the vehicle. The estimates are then updated with the gyroscope and accelerometer measurements. Since IMU measurements are used in the observation equation, this model can be easily modified to accommodate multiple IMUs. This approach is tested here. However, in terms of comparison with the other navigation solutions, the dynamics of the vehicle is considered unknown.

The fusion can also be done at the INS level. One approach used to fuse multiple INSs involves stacking their error states in a centralized structure [34,39]. However, with this method, if only one GPS is used, each INS error is corrected with the same GPS measurements. Therefore, the GPS observations for the different INSs are 100% correlated and lead to filter divergence. This problem can be solved by neglecting the inter-INS covariance in the Kalman filter observation covariance matrix. If no error states are shared among INSs, doing so is equivalent to consider all INSs within the stacked filter as independent navigation solutions [34,39]. Having the error states of all INSs in one model allows the exploitation of geometrical constraints, which can be the relative attitude, velocity and position between the IMUs [32,33,34,39]. These soft constraints are implemented as observations of almost perfect measurements [42]. However, the tuning of this navigation filter can be complex. For example, the relative velocity and position constraint variances must take into account the fact that their measurements are affected by the attitude estimation error [39]. Additionally, geometrical constraints cause inter-INS correlation accumulation. Considering that constraints do not provide absolute measurements but only relative values between the INSs, excessive inter-INS correlation must be avoided to prevent the navigation filter divergence. This can be done, among other things, by adjusting the geometrical constraint update rate [39]. The fusion of multiple INSs aided by a GPS and geometrical constraints has successfully been tested in the contexts of pedestrian navigation [34,39] and road vehicle navigation [43]. Many other approaches, such as the multiple versions of the federated filter [9,34], can be taken to perform the fusion at the INS level. However, the ability to include geometrical constraints makes the stacked filter interesting in a satellite launcher context, where the IMU relative positions along the vehicle structure are constant. Therefore, this one is selected here.

The objectives of this paper are to

- compare the performances of three IMUs against that of one better quality IMU;
- investigate the effect of collocating IMUs versus distributing them along the launcher structure;
- test three multi-IMU navigation solutions:
  – fusion of all IMUs in one INS,
  – fusion of multiple INSs, and
  – fusion of multiple INSs with geometrical constraints.

All navigation approaches are tested with and without a GPS receiver, while a three-axis attitude reference sensor is included in all navigation solutions. For the sake of generalization, the attitude sensors are generic and provide measurements only affected by white noises. The navigation fusion architectures are presented in Section 2. Section 3 introduces the test parameters, and results are analyzed in Section 4.

## 2. Data Fusion Architectures

All navigation solutions are implemented with a loosely coupled GPS and a three-axis attitude reference sensor. For the tests without the GPS receiver, the terms associated with the GPS velocity and position measurements are set to 0 in the observation equation of the navigation fusion. Section 2.1 presents the navigation fusion with a single IMU. Section 2.2 introduces the fusion of multiple IMUs in one INS. The fusion of multiple INSs in a stacked filter, with and without geometrical constraints, is presented in Section 2.3.

### 2.1. Fusion with One IMU

The first navigation solution uses only one IMU and is the baseline against which the other navigation solutions are compared. The error state vector includes the attitude $\delta \Psi_{e}^{E}$, velocity $\delta v_{e}^{E}$, and position $\delta r_{e}^{E}$ error estimations along with the gyroscope $b_{g}^{B}$, accelerometer $b_{a}^{B}$, and GPS position $b_{p}^{E}$ bias estimations. The navigation fusion model is

(1) $$\begin{array}{c} \left[\begin{array}{c} {\delta \Psi_{e}^{E}}_{\left(k+1\right)}\\ {\delta v_{e}^{E}}_{\left(k+1\right)}\\ {\delta r_{e}^{E}}_{\left(k+1\right)}\\ {b_{g}^{B}}_{\left(k+1\right)}\\ {b_{a}^{B}}_{\left(k+1\right)}\\ {b_{p}^{E}}_{\left(k+1\right)} \end{array}\right]=\left[\begin{array}{c} I_{18}+s_{t}\left[\begin{array}{cccccc} -\omega_{\mathrm{IE}}^{E}\times & 0_{3} & 0_{3} & -{T_{B}^{E}}_{\left(k\right)} & 0_{3} & 0_{3}\\ {T_{B}^{E}}_{\left(k\right)}{a_{m}^{B}}_{\left(k\right)}\times & -2\omega_{\mathrm{IE}}^{E}\times & 0_{3} & 0_{3} & {T_{B}^{E}}_{\left(k\right)} & 0_{3}\\ 0_{3} & I_{3} & 0_{3} & 0_{3} & 0_{3} & 0_{3}\\ 0_{3} & 0_{3} & 0_{3} & -\frac{1}{c_{g}}I_{3} & 0_{3} & 0_{3}\\ 0_{3} & 0_{3} & 0_{3} & 0_{3} & -\frac{1}{c_{a}}I_{3} & 0_{3}\\ 0_{3} & 0_{3} & 0_{3} & 0_{3} & 0_{3} & -\frac{1}{c_{p}}I_{3} \end{array}\right] \end{array}\right]\left[\begin{array}{c} {\delta \Psi_{e}^{E}}_{\left(k\right)}\\ {\delta v_{e}^{E}}_{\left(k\right)}\\ {\delta r_{e}^{E}}_{\left(k\right)}\\ {b_{g}^{B}}_{\left(k\right)}\\ {b_{a}^{B}}_{\left(k\right)}\\ {b_{p}^{E}}_{\left(k\right)} \end{array}\right]=\\ +s_{t}\left[\begin{array}{ccccc} -{T_{B}^{E}}_{\left(k\right)} & 0_{3} & 0_{3} & 0_{3} & 0_{3}\\ 0_{3} & {T_{B}^{E}}_{\left(k\right)} & 0_{3} & 0_{3} & 0_{3}\\ 0_{3} & 0_{3} & 0_{3} & 0_{3} & 0_{3}\\ 0_{3} & 0_{3} & \sqrt{\frac{2}{c_{g}s_{t}}}I_{3} & 0_{3} & 0_{3}\\ 0_{3} & 0_{3} & 0_{3} & \sqrt{\frac{2}{c_{a}s_{t}}}I_{3} & 0_{3}\\ 0_{3} & 0_{3} & 0_{3} & 0_{3} & \sqrt{\frac{2}{c_{p}s_{t}}}I_{3} \end{array}\right]\left[\begin{array}{c} {{\left[{\Delta \omega }_{m}\right]}_{\mathrm{IB}}^{B}}_{\left(k\right)}\\ {{\Delta a}_{m}^{B}}_{\left(k\right)}\\ {\Delta b_{g}^{B}}_{\left(k\right)}\\ {\Delta b_{a}^{B}}_{\left(k\right)}\\ {\Delta b_{p}^{E}}_{\left(k\right)} \end{array}\right] \end{array}$$

(2) $$\begin{array}{cc} \left[\begin{array}{c} {\delta \Psi_{m}^{E}}_{\left(k\right)}\\ {\delta v_{m}^{E}}_{\left(k\right)}\\ {\delta r_{m}^{E}}_{\left(k\right)} \end{array}\right] & =\left[\begin{array}{cccccc} I_{3} & 0_{3} & 0_{3} & 0_{3} & 0_{3} & 0_{3}\\ 0_{3} & I_{3} & 0_{3} & 0_{3} & 0_{3} & 0_{3}\\ 0_{3} & 0_{3} & I_{3} & 0_{3} & 0_{3} & I_{3} \end{array}\right]\left[\begin{array}{c} {\delta \Psi_{e}^{E}}_{\left(k\right)}\\ {\delta v_{e}^{E}}_{\left(k\right)}\\ {\delta r_{e}^{E}}_{\left(k\right)}\\ {b_{g}^{B}}_{\left(k\right)}\\ {b_{a}^{B}}_{\left(k\right)}\\ {b_{p}^{E}}_{\left(k\right)} \end{array}\right]+\left[\begin{array}{c} {\Delta \Psi_{m}^{E}}_{\left(k\right)}\\ {\Delta v_{m}^{E}}_{\left(k\right)}\\ {\Delta r_{m}^{E}}_{\left(k\right)} \end{array}\right] \end{array}$$

where $0_{i}$ is a i×i zero matrix, and $I_{i}$ is a i×i identity matrix. The biases are modeled by Markov processes with correlation time $c_{g}$ for the gyroscopes, $c_{a}$ for the accelerometers, and $c_{p}$ for the GPS position. The estimated rotation matrix from the body frame to the Earth frame is $T_{B}^{E}$, and the Earth rate is $\omega_{\mathrm{IE}}^{E}$ (i.e., the rotation of the Earth frame *E* with respect to the inertial frame *I*). The sampling time is $s_{t}$, and *k* is the time step. The attitude, velocity, and position error measurements are, respectively, $\delta \Psi_{m}^{E}$, $\delta v_{m}^{E}$, and $\delta r_{m}^{E}$, and their corresponding noises are $\Delta \Psi_{m}^{E}$, $\Delta v_{m}^{E}$, and $\Delta r_{m}^{E}$. The superscripts ${\left\{\cdot \right\}}^{E}$ and ${\left\{\cdot \right\}}^{B}$ mean that the variable is represented in the Earth and body frame, respectively.

### 2.2. Fusion of Multiple IMUs within One INS

In order to fuse multiple IMUs, their measurements must be transformed in a common position. If the structure is rigid, and all IMUs aligned with the body frame, all gyroscopes within the same axis experience the same angular velocity. However, the specific forces sensed by the accelerometers depend on the relative position of the IMUs [35,36,37]. The transformation of accelerometer measurements to the common position is referred to as the Grubin transformation [36] within this paper. To perform the Grubin transformation, the angular acceleration ${\left[\alpha_{e}\right]}_{\mathrm{IB}}^{B}$ is required [36,39]. Since it is not measured, it will be estimated within the navigation filter. If all IMUs are identical, the average of the *N* IMU measurements transformed in the common position can be used as the input for the INS. The average of the gyroscope measurements is

(3) $$\begin{array}{c} {{\left[\omega_{\mathrm{me}}\right]}_{\mathrm{IB}}^{B}}_{\left(k\right)}=\frac{1}{N}\sum_{n=1}^{N}{{\left[\omega_{m_{n}}\right]}_{\mathrm{IB}}^{B}}_{\left(k\right)} \end{array}$$

where ${{\left[\omega_{m_{n}}\right]}_{\mathrm{IB}}^{B}}_{\left(k\right)}$ is the gyroscope measurement of the $n^{\mathrm{th}}$ IMU. The average of the accelerometer measurements is

(4) $$\begin{array}{c} a_{\mathrm{me}}^{B}=\frac{1}{N}\sum_{n=1}^{N}\left(a_{m_{n}}^{B}-{{\left[\omega_{\mathrm{me}}\right]}_{\mathrm{IB}}^{B}}_{\left(k\right)}\times {{\left[\omega_{\mathrm{me}}\right]}_{\mathrm{IB}}^{B}}_{\left(k\right)}\times l_{n}^{B}-{{\left[\alpha_{e}\right]}_{\mathrm{IB}}^{B}}_{\left(k\right)}\times l_{n}^{B}\right) \end{array}$$

where $a_{m_{n}}^{B}$ is the accelerometer measurement of the $n^{\mathrm{th}}$ IMU. The lever arm between the $n^{\mathrm{th}}$ IMU position and the common position is $l_{n}^{B}$.

The navigation fusion model comes from Beaudoin et al. [41]. However, in the present work, the launcher dynamics is considered completely unknown, and multiple gyroscope and accelerometer measurements are included in the observation equation. The navigation fusion propagation equation is

(5) $$\begin{array}{cc} \;\left[\begin{array}{c} {{\left[\alpha_{e}\right]}_{\mathrm{IB}}^{B}}_{\left(k+1\right)}\\ {{\left[\omega_{e}\right]}_{\mathrm{IB}}^{B}}_{\left(k+1\right)}\\ {a_{e}^{B}}_{\left(k+1\right)}\\ {\delta \Psi_{e}^{E}}_{\left(k+1\right)}\\ {\delta v_{e}^{E}}_{\left(k+1\right)}\\ {\delta r_{e}^{E}}_{\left(k+1\right)}\\ {b_{g_{1}}^{B}}_{\left(k+1\right)}\\ \vdots \\ {b_{g_{N}}^{B}}_{\left(k+1\right)}\\ {b_{a_{1}}^{B}}_{\left(k+1\right)}\\ \vdots \\ {b_{a_{N}}^{B}}_{\left(k+1\right)}\\ {b_{p}^{E}}_{\left(k+1\right)} \end{array}\right] & =\left[\begin{array}{c} {{\left[\alpha_{e}\right]}_{\mathrm{IB}}^{B}}_{\left(k\right)}\\ {{\left[\omega_{e}\right]}_{\mathrm{IB}}^{B}}_{\left(k\right)}\\ {a_{e}^{B}}_{\left(k\right)}\\ {\delta \Psi_{e}^{E}}_{\left(k\right)}\\ {\delta v_{e}^{E}}_{\left(k\right)}\\ {\delta r_{e}^{E}}_{\left(k\right)}\\ {b_{g_{1}}^{B}}_{\left(k\right)}\\ \vdots \\ {b_{g_{N}}^{B}}_{\left(k\right)}\\ {b_{a_{1}}^{B}}_{\left(k\right)}\\ \vdots \\ {b_{a_{N}}^{B}}_{\left(k\right)}\\ {b_{p}^{E}}_{\left(k\right)} \end{array}\right]+s_{t}\left[\begin{array}{c} {{\left[{\Delta \alpha }_{e}\right]}_{\mathrm{IB}}^{B}}_{\left(k\right)}\\ {{\left[\alpha_{e}\right]}_{\mathrm{IB}}^{B}}_{\left(k\right)}\\ {{\Delta a}_{e}^{B}}_{\left(k\right)}\\ {-T_{B}^{E}}_{\left(k\right)}\left({{\left[\omega_{\mathrm{me}}\right]}_{\mathrm{IB}}^{B}}_{\left(k\right)}-{{\left[\omega_{e}\right]}_{\mathrm{IB}}^{B}}_{\left(k\right)}\right)-\omega_{\mathrm{IE}}^{E}\times {\delta \Psi_{e}^{E}}_{\left(k\right)}\\ {T_{B}^{E}}_{\left(k\right)}\left({a_{\mathrm{me}}^{B}}_{\left(k\right)}-{a_{e}^{B}}_{\left(k\right)}\right)+{T_{B}^{E}}_{\left(k\right)}{a_{\mathrm{me}}^{B}}_{\left(k\right)}\times {\delta \Psi_{e}^{E}}_{\left(k\right)}-2\omega_{\mathrm{IE}}^{E}\times {\delta v_{e}^{E}}_{\left(k\right)}\\ {\delta v_{e}^{E}}_{\left(k\right)}\\ -\frac{1}{c_{g}}{b_{g_{1}}^{B}}_{\left(k\right)}+\sqrt{\frac{2}{c_{g}s_{t}}}{\Delta b_{g_{1}}^{B}}_{\left(k\right)}\\ \vdots \\ -\frac{1}{c_{g}}{b_{g_{N}}^{B}}_{\left(k\right)}+\sqrt{\frac{2}{c_{g}s_{t}}}{\Delta b_{g_{N}}^{B}}_{\left(k\right)}\\ -\frac{1}{c_{a}}{b_{a_{1}}^{B}}_{\left(k\right)}+\sqrt{\frac{2}{c_{a}s_{t}}}{\Delta b_{a_{1}}^{B}}_{\left(k\right)}\\ \vdots \\ -\frac{1}{c_{a}}{b_{a_{N}}^{B}}_{\left(k\right)}+\sqrt{\frac{2}{c_{a}s_{t}}}{\Delta b_{a_{N}}^{B}}_{\left(k\right)}\\ -\frac{1}{c_{p}}{b_{p}^{E}}_{\left(k\right)}+\sqrt{\frac{2}{c_{a}s_{t}}}{\Delta b_{p}^{E}}_{\left(k\right)} \end{array}\right] \end{array}$$

where the acceleration estimation is $a_{e}^{B}$, the jerk estimation is ${\Delta a}_{e}^{B}$, the angular velocity estimation is ${\left[\omega_{e}\right]}_{\mathrm{IB}}^{B}$, and the estimated change rate of the angular acceleration is ${\left[{\Delta \alpha }_{e}\right]}_{\mathrm{IB}}^{B}$. The gyroscope bias estimations are $b_{g_{1}}^{B},\cdots ,b_{g_{N}}^{B}$, the accelerometer bias estimations are $b_{a_{1}}^{B},\cdots ,b_{a_{N}}^{B}$. The navigation fusion observation equation is

(6) $$\begin{array}{cc} \left[\begin{array}{c} {{\left[\omega_{m_{1}}\right]}_{\mathrm{IB}}^{B}}_{\left(k\right)}\\ \vdots \\ {{\left[\omega_{m_{N}}\right]}_{\mathrm{IB}}^{B}}_{\left(k\right)}\\ {a_{m_{1}}^{B}}_{\left(k\right)}\\ \vdots \\ {a_{m_{N}}^{B}}_{\left(k\right)}\\ {\delta \Psi_{m}^{E}}_{\left(k\right)}\\ {\delta v_{m}^{E}}_{\left(k\right)}\\ {\delta r_{m}^{E}}_{\left(k\right)} \end{array}\right] & =\left[\begin{array}{c} {{\left[\omega_{e}\right]}_{\mathrm{IB}}^{B}}_{\left(k\right)}+{b_{g_{1}}^{B}}_{\left(k\right)}+{{\left[{\Delta \omega }_{m_{1}}\right]}_{\mathrm{IB}}^{B}}_{\left(k\right)}\\ \vdots \\ {{\left[\omega_{e}\right]}_{\mathrm{IB}}^{B}}_{\left(k\right)}+{b_{g_{N}}^{B}}_{\left(k\right)}+{{\left[{\Delta \omega }_{m_{N}}\right]}_{\mathrm{IB}}^{B}}_{\left(k\right)}\\ {a_{e}^{B}}_{\left(k\right)}+{b_{a_{1}}^{B}}_{\left(k\right)}+{{\Delta a}_{m_{1}}^{B}}_{\left(k\right)}+l_{1}^{B}\times {{\left[\alpha_{e}\right]}_{\mathrm{IB}}^{B}}_{\left(k\right)}+{G_{1}}_{\left(k\right)}{{\left[\omega_{e}\right]}_{\mathrm{IB}}^{B}}_{\left(k\right)}\\ \vdots \\ {a_{e}^{B}}_{\left(k\right)}+{b_{a_{N}}^{B}}_{\left(k\right)}+{{\Delta a}_{m_{N}}^{B}}_{\left(k\right)}+l_{N}^{B}\times {{\left[\alpha_{e}\right]}_{\mathrm{IB}}^{B}}_{\left(k\right)}+{G_{N}}_{\left(k\right)}{{\left[\omega_{e}\right]}_{\mathrm{IB}}^{B}}_{\left(k\right)}\\ {\delta \Psi_{e}^{E}}_{\left(k\right)}+{\Delta \Psi_{m}^{E}}_{\left(k\right)}\\ {\delta v_{e}^{E}}_{\left(k\right)}+{\Delta v_{m}^{E}}_{\left(k\right)}\\ {\delta r_{e}^{E}}_{\left(k\right)}+{b_{p}^{E}}_{\left(k\right)}+{\Delta r_{m}^{E}}_{\left(k\right)} \end{array}\right] \end{array}$$

where

(7) $$\begin{array}{c} G_{n}=\left[{{\left[\omega_{\mathrm{me}}\right]}_{\mathrm{IB}}^{B}}_{\left(k\right)}\times \right]\left[l_{n}^{B}\times \right]+\left[\left({{\left[\omega_{\mathrm{me}}\right]}_{\mathrm{IB}}^{B}}_{\left(k\right)}\times l_{n}^{B}\right)\times \right],\forall n\in \left\{1,\ldots ,N\right\}. \end{array}$$

**Figure 1:**
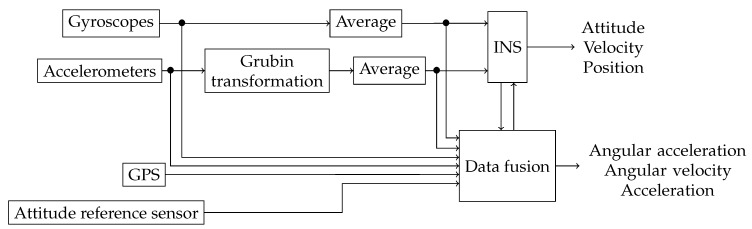
Structure of the fusion of multiple IMUs in one INS.

The gyroscope ${\left[\omega_{m_{1}}\right]}_{\mathrm{IB}}^{B},\cdots ,{\left[\omega_{m_{N}}\right]}_{\mathrm{IB}}^{B}$ and accelerometer $a_{m_{1}}^{B},\cdots ,a_{m_{N}}^{B}$ measurements are affected by their corresponding noises ${\left[{\Delta \omega }_{m_{1}}\right]}_{\mathrm{IB}}^{B},\cdots ,{\left[{\Delta \omega }_{m_{N}}\right]}_{\mathrm{IB}}^{B}$, and ${\Delta a}_{m_{1}}^{B},\cdots ,{\Delta a}_{m_{N}}^{B}$. Figure 1 summarizes the structure of the navigation, which fuses multiple IMUs within one INS.

### 2.3. Fusion of Multiple INSs in a Stacked Filter

The fusion of multiple INSs is done by combining the error states of each INS in a block diagonal structure [43]. The propagation equation of the navigation model is

(8) $$\begin{array}{c} \left[\begin{array}{c} {x_{{\mathrm{INS}}_{1}}^{}}_{\left(k+1\right)}\\ {x_{{\mathrm{INS}}_{2}}^{}}_{\left(k+1\right)}\\ \vdots \\ {x_{{\mathrm{INS}}_{N}}^{}}_{\left(k+1\right)} \end{array}\right]=\left[\begin{array}{cccc} {A_{INS_{1}}}_{\left(k\right)} & 0 & \cdots & 0\\ 0 & {A_{INS_{2}}}_{\left(k\right)} & \cdots & 0\\ \vdots & \vdots & \ddots & \vdots \\ 0 & 0 & \cdots & {A_{INS_{N}}}_{\left(k\right)} \end{array}\right]\left[\begin{array}{c} {x_{{\mathrm{INS}}_{1}}^{}}_{\left(k\right)}\\ {x_{{\mathrm{INS}}_{2}}^{}}_{\left(k\right)}\\ \vdots \\ {x_{{\mathrm{INS}}_{N}}^{}}_{\left(k\right)} \end{array}\right]\\ +\left[\begin{array}{cccc} {B_{INS_{1}}}_{\left(k\right)} & 0 & \cdots & 0\\ 0 & {B_{INS_{2}}}_{\left(k\right)} & \cdots & 0\\ \vdots & \vdots & \ddots & \vdots \\ 0 & 0 & \cdots & {B_{INS_{N}}}_{\left(k\right)} \end{array}\right]\left[\begin{array}{c} {w_{{\mathrm{INS}}_{1}}^{}}_{\left(k\right)}\\ {w_{{\mathrm{INS}}_{2}}^{}}_{\left(k\right)}\\ \vdots \\ {w_{{\mathrm{INS}}_{N}}^{}}_{\left(k\right)} \end{array}\right] \end{array}$$

where $x_{{\mathrm{INS}}_{1}}^{},\cdots ,x_{{\mathrm{INS}}_{N}}^{}$ are the error state vectors of the *N* INSs. The matrices $A_{INS_{1}},\cdots ,A_{INS_{N}}$, and $B_{INS_{1}},\cdots ,B_{INS_{N}}$ represent the state and input matrices of the individual INSs, and $w_{{\mathrm{INS}}_{1}}^{},\cdots ,w_{{\mathrm{INS}}_{N}}^{}$ represents the corresponding input noise vectors. The observation equation is

(9) $$\begin{array}{cc} \left[\begin{array}{c} {y_{{\mathrm{INS}}_{1}}^{}}_{\left(k\right)}\\ {y_{{\mathrm{INS}}_{2}}^{}}_{\left(k\right)}\\ \vdots \\ {y_{{\mathrm{INS}}_{N}}^{}}_{\left(k\right)} \end{array}\right] & =\left[\begin{array}{cccc} {C_{INS_{1}}}_{\left(k\right)} & 0 & \cdots & 0\\ 0 & {C_{INS_{2}}}_{\left(k\right)} & \cdots & 0\\ \vdots & \vdots & \ddots & \vdots \\ 0 & 0 & \cdots & {C_{INS_{N}}}_{\left(k\right)} \end{array}\right]\left[\begin{array}{c} {x_{{\mathrm{INS}}_{1}}^{}}_{\left(k\right)}\\ {x_{{\mathrm{INS}}_{2}}^{}}_{\left(k\right)}\\ \vdots \\ {x_{{\mathrm{INS}}_{N}}^{}}_{\left(k\right)} \end{array}\right]+\left[\begin{array}{c} {v_{{\mathrm{INS}}_{1}}^{}}_{\left(k\right)}\\ {v_{{\mathrm{INS}}_{2}}^{}}_{\left(k\right)}\\ \vdots \\ {v_{{\mathrm{INS}}_{N}}^{}}_{\left(k\right)} \end{array}\right] \end{array}$$

where $y_{{\mathrm{INS}}_{1}}^{},\cdots ,y_{{\mathrm{INS}}_{N}}^{}$ are the output vectors, $C_{INS_{1}},\cdots ,C_{INS_{N}}$ the output matrices, and $v_{{\mathrm{INS}}_{1}}^{},\cdots ,v_{{\mathrm{INS}}_{N}}^{}$ the output noise vectors. The error state vectors can be different for each INS, and if no error states are shared among INSs, the stacked filter hence created is equivalent to *N* independent navigation filters. This arrangement allows for the addition of geometrical constraints on the relative attitude, velocity, and position between the IMUs. These constraints are included in the model as additional observations (Section 2.3.1). The stacked filter provides *N* estimations of the navigation states. These must be combined to obtain the final navigation solution (Section 2.3.2). The INS error state models used in the stacked filter for this research are presented in Section 2.3.3.

#### 2.3.1. Geometrical Constraint Equations

If all IMUs are aligned with the body frame, the real attitude $\Psi_{r_{n}}^{E}$ of the *N* IMUs are equal:

(10) $$\begin{array}{c} {\Psi_{r_{n}}^{E}}_{\left(k\right)}={\Psi_{r_{m}}^{E}}_{\left(k\right)},\forall n,m\in \left\{1,\cdots ,N\right\}. \end{array}$$

the attitude estimation $\Psi_{e_{n}}^{E}$ of the $n^{\mathrm{th}}$ INS is

(11) $$\begin{array}{cc} {\Psi_{e_{n}}^{E}}_{\left(k\right)} & ={\Psi_{r_{n}}^{E}}_{\left(k\right)}+{\delta \Psi_{e_{n}}^{E}}_{\left(k\right)} \end{array}$$

where $\delta \Psi_{e_{n}}^{E}$ is the attitude estimation error of the $n^{\mathrm{th}}$ INS. Subtracting the attitude estimation of the $m^{\mathrm{th}}$ INS from the attitude estimation of the $n^{\mathrm{th}}$ INS, and using Equation (10) gives

(12) $$\begin{array}{c} {\Psi_{e_{n}}^{E}}_{\left(k\right)}-{\Psi_{e_{m}}^{E}}_{\left(k\right)}={\Psi_{r_{n}}^{E}}_{\left(k\right)}+{\delta \Psi_{e_{n}}^{E}}_{\left(k\right)}-{\Psi_{r_{m}}^{E}}_{\left(k\right)}-{\delta \Psi_{e_{m}}^{E}}_{\left(k\right)} \end{array}$$

(13) $$\begin{array}{c} ={\Psi_{r_{n}}^{E}}_{\left(k\right)}+{\delta \Psi_{e_{n}}^{E}}_{\left(k\right)}-{\Psi_{r_{n}}^{E}}_{\left(k\right)}-{\delta \Psi_{e_{m}}^{E}}_{\left(k\right)} \end{array}$$

(14) $$\begin{array}{c} ={\delta \Psi_{e_{n}}^{E}}_{\left(k\right)}-{\delta \Psi_{e_{m}}^{E}}_{\left(k\right)}, \end{array}$$

which is the observation equation of the relative attitude between the $n^{\mathrm{th}}$ INS and the $m^{\mathrm{th}}$ INS.

The relation between the real velocity $v_{r_{n}}^{E}$ of the $n^{\mathrm{th}}$ INS and the real velocity $v_{r_{m}}^{E}$ of the $m^{\mathrm{th}}$ INS [37] is

(15) $$\begin{array}{c} {v_{r_{n}}^{E}}_{\left(k\right)}={v_{r_{m}}^{E}}_{\left(k\right)}+{{\left[T_{r}\right]}_{B}^{E}}_{\left(k\right)}\left({{\left[\omega_{r}\right]}_{\mathrm{EB}}^{B}}_{\left(k\right)}l_{n,m}^{B}\right),\forall n,m\in \left\{1,\cdots ,N\right\} \end{array}$$

where $l_{n,m}^{B}$ is the lever arm between the two INSs, ${{\left[\omega_{r}\right]}_{\mathrm{EB}}^{B}}_{\left(k\right)}$ is the angular velocity of the body frame with respect to the Earth frame, and ${\left[T_{r}\right]}_{B}^{E}$ is the real rotation matrix from the body to the Earth frame. The velocity estimation ${v_{e_{n}}^{E}}_{\left(k\right)}$ of the $n^{\mathrm{th}}$ INS is

(16) $$\begin{array}{cc} {v_{e_{n}}^{E}}_{\left(k\right)} & ={v_{r_{n}}^{E}}_{\left(k\right)}+{\delta v_{e_{n}}^{E}}_{\left(k\right)} \end{array}$$

where $\delta v_{e_{n}}^{E}$ is the velocity estimation error of the $n^{\mathrm{th}}$ INS. Subtracting the velocity estimation of the $m^{\mathrm{th}}$ INS from the $n^{\mathrm{th}}$ INS one,

(17) $$\begin{array}{cc} {v_{e_{n}}^{E}}_{\left(k\right)}-{v_{e_{m}}^{E}}_{\left(k\right)} & ={v_{r_{n}}^{E}}_{\left(k\right)}+{\delta v_{e_{n}}^{E}}_{\left(k\right)}-{v_{r_{m}}^{E}}_{\left(k\right)}-{\delta v_{e_{m}}^{E}}_{\left(k\right)}, \end{array}$$

inserting Equation (15),

(18) $$\begin{array}{c} {v_{e_{n}}^{E}}_{\left(k\right)}-{v_{e_{m}}^{E}}_{\left(k\right)}={v_{r_{m}}^{E}}_{\left(k\right)}+{{\left[T_{r}\right]}_{B}^{E}}_{\left(k\right)}\left({{\left[\omega_{r}\right]}_{\mathrm{EB}}^{B}}_{\left(k\right)}l_{n,m}^{B}\right)+{\delta v_{e_{n}}^{E}}_{\left(k\right)}-{v_{r_{m}}^{E}}_{\left(k\right)}-{\delta v_{e_{m}}^{E}}_{\left(k\right)} \end{array}$$

(19) $$\begin{array}{c} ={{\left[T_{r}\right]}_{B}^{E}}_{\left(k\right)}\left({{\left[\omega_{r}\right]}_{\mathrm{EB}}^{B}}_{\left(k\right)}l_{n,m}^{B}\right)+{\delta v_{e_{n}}^{E}}_{\left(k\right)}-{\delta v_{e_{m}}^{E}}_{\left(k\right)}, \end{array}$$

and rearranging terms,

(20) $$\begin{array}{c} {v_{e_{n}}^{E}}_{\left(k\right)}-{v_{e_{m}}^{E}}_{\left(k\right)}-{{\left[T_{r}\right]}_{B}^{E}}_{\left(k\right)}\left({{\left[\omega_{r}\right]}_{\mathrm{EB}}^{B}}_{\left(k\right)}l_{n,m}^{B}\right)={\delta v_{e_{n}}^{E}}_{\left(k\right)}-{\delta v_{e_{m}}^{E}}_{\left(k\right)}, \end{array}$$

yields the observation equation for the velocity constraint between the $n^{\mathrm{th}}$ INS and $m^{\mathrm{th}}$ INS. For the implementation, ${{\left[T_{r}\right]}_{B}^{E}}_{\left(k\right)}$ is replaced by its estimation ${T_{B}^{E}}_{\left(k\right)}$, and ${\left[\omega_{r}\right]}_{\mathrm{EB}}^{B}$ is replaced by ${\left[\omega_{\mathrm{me}}\right]}_{\mathrm{IB}}^{B}-\omega_{\mathrm{IE}}^{B}$:

(21) $$\begin{array}{c} {v_{e_{n}}^{E}}_{\left(k\right)}-{v_{e_{m}}^{E}}_{\left(k\right)}-{T_{B}^{E}}_{\left(k\right)}\left(\left({{\left[\omega_{\mathrm{me}}\right]}_{\mathrm{IB}}^{B}}_{\left(k\right)}-\omega_{\mathrm{IE}}^{B}\right)l_{n,m}^{B}\right)={\delta v_{e_{n}}^{E}}_{\left(k\right)}-{\delta v_{e_{m}}^{E}}_{\left(k\right)}. \end{array}$$

if the sensors are collocated, $l_{n,m}^{B}=0^{}$, which allows for the simplification of the relative velocity equation:

(22) $$\begin{array}{c} {v_{e_{n}}^{E}}_{\left(k\right)}-{v_{e_{m}}^{E}}_{\left(k\right)}={\delta v_{e_{n}}^{E}}_{\left(k\right)}-{\delta v_{e_{m}}^{E}}_{\left(k\right)}. \end{array}$$

The relation between the real position $r_{r_{n}}^{E}$ of the $n^{\mathrm{th}}$ INS and the real position $r_{r_{m}}^{E}$ of the $m^{\mathrm{th}}$ INS is given by

(23) $$\begin{array}{c} {r_{r_{n}}^{E}}_{\left(k\right)}={r_{r_{m}}^{E}}_{\left(k\right)}+{{\left[T_{r}\right]}_{B}^{E}}_{\left(k\right)}l_{n,m}^{B},\forall n,m\in \left\{1,\cdots ,N\right\}. \end{array}$$

the position estimation ${r_{e_{n}}^{E}}_{\left(k\right)}$ is

(24) $$\begin{array}{cc} {r_{e_{n}}^{E}}_{\left(k\right)} & ={r_{r_{n}}^{E}}_{\left(k\right)}+{\delta r_{e_{n}}^{E}}_{\left(k\right)} \end{array}$$

where ${\delta r_{e_{n}}^{E}}_{\left(k\right)}$ is the $n^{\mathrm{th}}$ INS position estimation error. Subtracting the $m^{\mathrm{th}}$ INS position estimation from the $n^{\mathrm{th}}$ INS one gives

(25) $$\begin{array}{cc} {r_{e_{n}}^{E}}_{\left(k\right)}-{r_{e_{m}}^{E}}_{\left(k\right)} & ={r_{r_{n}}^{E}}_{\left(k\right)}+{\delta r_{e_{n}}^{E}}_{\left(k\right)}-{r_{r_{m}}^{E}}_{\left(k\right)}-{\delta r_{e_{m}}^{E}}_{\left(k\right)}. \end{array}$$

inserting Equation (23) into Equation (25) and rearranging terms yield the observation equation for the position constraint between the $n^{\mathrm{th}}$ and $m^{\mathrm{th}}$ INSs:

(26) $$\begin{array}{cc} {r_{e_{n}}^{E}}_{\left(k\right)}-{r_{e_{m}}^{E}}_{\left(k\right)}-{{\left[T_{r}\right]}_{B}^{E}}_{\left(k\right)}l_{n,m}^{B} & ={\delta r_{e_{n}}^{E}}_{\left(k\right)}-{\delta r_{e_{m}}^{E}}_{\left(k\right)}, \end{array}$$

which, with the available estimated values, gives

(27) $$\begin{array}{cc} {r_{e_{n}}^{E}}_{\left(k\right)}-{r_{e_{m}}^{E}}_{\left(k\right)}-{T_{B}^{E}}_{\left(k\right)}l_{n,m}^{B} & ={\delta r_{e_{n}}^{E}}_{\left(k\right)}-{\delta r_{e_{m}}^{E}}_{\left(k\right)}. \end{array}$$

as with the relative velocity constraints, when the sensors are collocated, the relative position equation can be simplified as

(28) $$\begin{array}{cc} {r_{e_{n}}^{E}}_{\left(k\right)}-{r_{e_{m}}^{E}}_{\left(k\right)} & ={\delta r_{e_{n}}^{E}}_{\left(k\right)}-{\delta r_{e_{m}}^{E}}_{\left(k\right)}. \end{array}$$

Using the relative attitude, velocity, and position equations for all pairs of INSs yields the observations needed to add the geometrical constraints to the data fusion model.

#### 2.3.2. Fusion of all INSs

**Figure 2:**
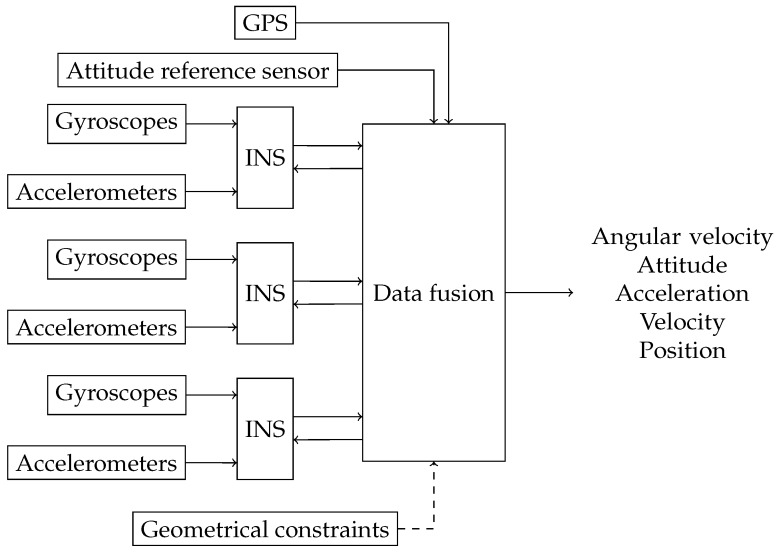
Fusion structure of three INSs.

The final step, regardless of whether the geometrical constraints are present or not, is to combine the *N* INS estimates. To do so, each INS estimate is transformed in the common position using the Grubin transformation for the acceleration, Equation (15) for the velocity, and Equation (23) for the position. Since all IMUs are aligned with the body frame, the angular velocity and attitude do not need transformation. The average of the INS estimates in the common position is then used as the final navigation solution. Figure 2 summarizes the structure for the fusion of three INSs.

#### 2.3.3. Individual INS Error State Models

The error state models of the INSs within the stacked filter are based on the one used for the single IMU presented in Section 2.1. However, to reduce the size of the stacked filter model, one common GPS position bias $b_{p}^{E}$ is estimated for all INSs (i.e., only Equation (1) of the first INS includes $b_{p}^{E}$, which is shared among all INSs in Equation (2)). Therefore, the first INS has 18 error states and the other INSs have 15 error states. In Section 4.1, it will be shown that, in addition to reducing the size of the model, considering one GPS position bias estimation provides performance improvement over estimating the GPS position bias independently for each INS.

Since only one GPS receiver and one attitude reference sensor are used, their measurements are 100% correlated among the INSs. As stated in the introduction, to avoid filter divergence, the inter-block covariances for the GPS and attitude reference sensor must be neglected in the Kalman filter observation covariance matrix.

## 3. Test Parameters

**Figure 3:**
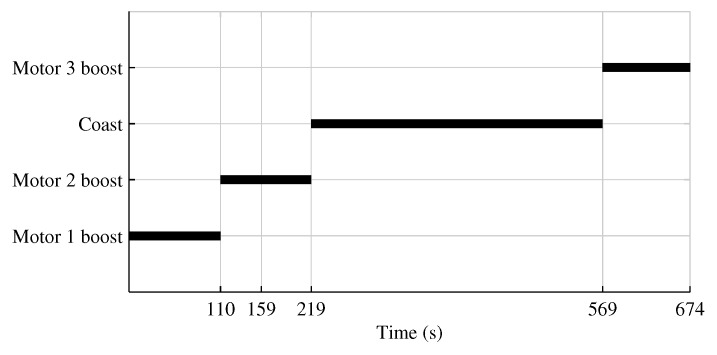
Mission phase timeline.

The non-linear launcher simulator used in this research is provided by Defence Research and Development Canada. The launcher dynamics model is based on Zipfel [36], while the guidance is performed using the Schuler approximation [44] and the control is done with a cascade PI controller [45]. Among other things, this simulator considers the wind and aerodynamic coefficients, which vary with the velocity, altitude, and aerodynamic angles of the vehicle. The simulated mission is intended to put a satellite on a sun-synchronous orbit at an altitude of 500 km. The launch is performed from Churchill, Manitoba, Canada. The complete mission time is 674 s. Figure 3 summarizes the mission phase timeline.

The sensor specifications are given in Table 1. The IMU specifications for the multi-IMU configurations are inspired by the IMU-KVH1750 unit from Novatel${}^{®}$. To obtain the same volume of information from the sensors among the navigation solutions, the specifications for the single IMU configuration are divided by $\sqrt{3}$ in comparison to the multi-IMU solutions.

To simplify the implementation and without loss of generality, the update rate of the GPS, of the attitude reference sensor, and of the geometrical constraints is set to 1 s. Additionally, the launcher is modeled as a rigid structure.

To ensure that they are available throughout the duration of the mission, the GPS and the attitude reference sensor are always installed in the launcher head, as are the IMUs in the collocated and single IMU configurations. Obviously, perfectly collocated sensors are impossible to implement. However, this configuration is interesting as a comparison basis. For the distributed IMUs, one IMU is installed at the top of each of the three launcher stages. The head of the launcher stages is selected to maximize the distance between the IMUs while avoiding having IMUs inside motors. The tops of the lower stages are, respectively, at 2.8 and 14.6 m from the launcher head.

The variance of the geometrical constraints was set experimentally. Good results were obtained with 1 ${{(}^{{}^{\circ}})}^{2}$, 1 (m/s)${}^{2}$, and 1 m${}^{2}$ for the attitude, velocity, and position constraints, respectively. For each test, the time evolution of the standard deviations estimated by the navigation fusion is used as a comparison basis. To ensure that the theoretical standard deviations correspond to the real ones, all results are verified with a 10-run Monte Carlo simulation. To clarify the graphics that present standard deviations (i.e., to avoid the sawtooth-shape), the values are sampled at 1 Hz, before the GPS, attitude reference sensor, and geometrical constraint update. Therefore, they represent the worst navigation precision for every second of simulation.

## 4. Results Analysis

### 4.1. Tests with All Sensors in the Launcher Head

In this section, all sensors are installed in the launcher head. The main advantage of having the sensors at this location is that no sensors are lost during the mission due to jettisoning. However, this configuration does not exploit the accelerometer measurements to improve the angular dynamics estimation.

#### 4.1.1. Test with GPS Receiver

**Figure 4:**
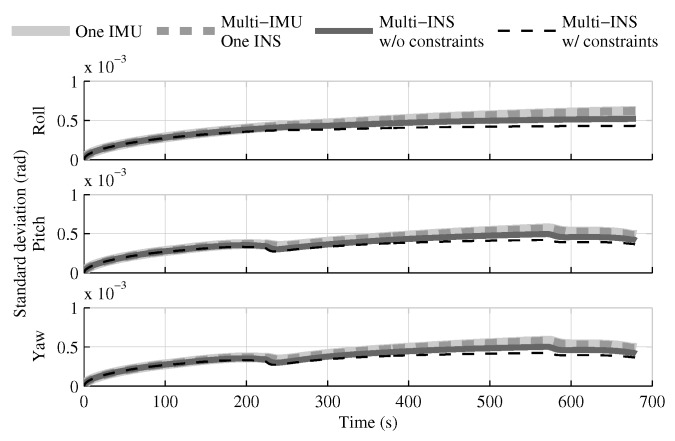
The attitude estimation error standard deviation obtained with the navigation solutions, when all sensor measurements are present.

**Figure 5:**
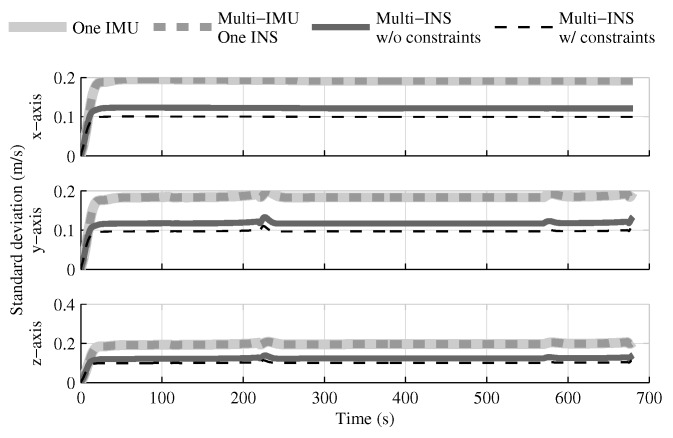
The velocity estimation error standard deviation obtained with the navigation solutions, when all sensor measurements are present.

**Figure 6:**
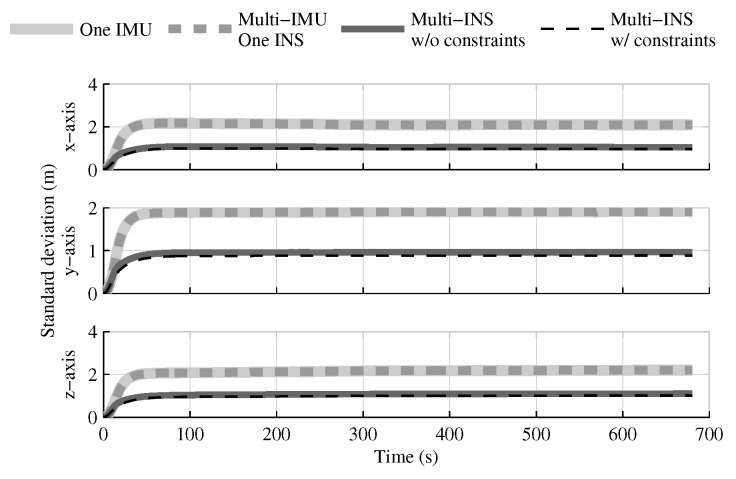
The position estimation error standard deviation obtained with the navigation solutions, when all sensor measurements are present.

When the GPS and attitude reference sensor are present, the solution which fuses all IMUs in one INS provides the same performances as in the single IMU approach (Figure 4, Figure 5 and Figure 6). This result was predictable, since with collocated IMUs, both approaches have access to the same volume of information [10,22]. The number of IMUs compensate for the lower quality sensors in the multi-IMU solution. Furthermore, it confirms that the solution proposed by the authors [41] performs as expected in a multi-IMU context.

**Figure 7:**
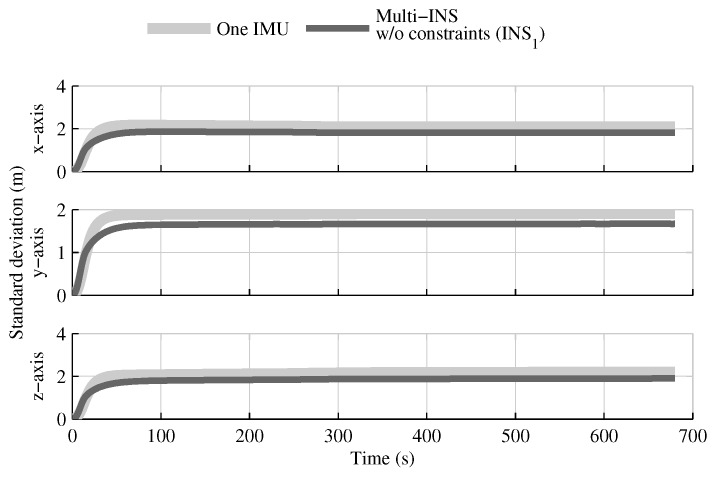
The position estimation error standard deviation obtained with the first INS of the multi-INS fusion without constraints versus the one obtained with the single IMU solution.

**Figure 8:**
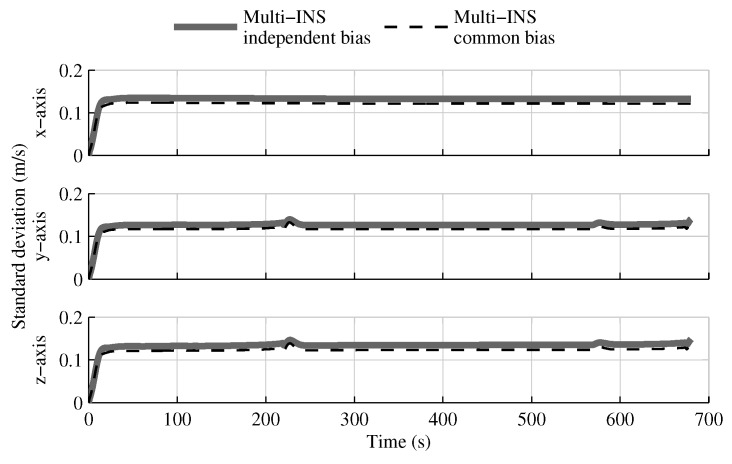
The velocity standard deviation obtained with a common GPS bias estimation versus one GPS bias estimated for each INS in the multi-INS fusion without constraint, when all sensor measurements are present.

**Figure 9:**
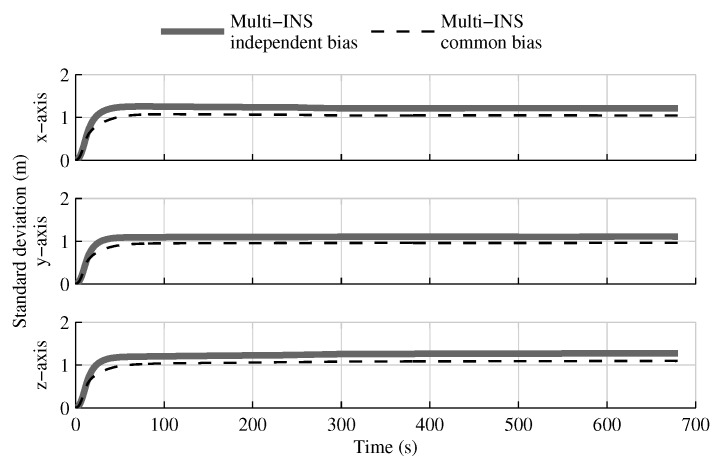
The position standard deviation obtained with a common GPS bias estimation versus one GPS bias estimated for each INS in the multi-INS fusion without constraint, when all sensor measurements are present.

The fusion of multiple INSs provides better estimates than the single IMU solution, even when the geometrical constraints are not present (Figure 4, Figure 5 and Figure 6). Adding the geometrical constraints further reduces the standard deviation of the estimates. The gains obtained are due, in part, to the fact that the fusion of multiple INSs makes better use of the available information by generating multiple estimations of the attitude, velocity, and position. Since each INS within the multi-INS fusion receives a lower volume of IMU information than in the single INS solutions (either with a single or multiple IMUs), a higher relative weight is put on the GPS and attitude reference sensor measurements. Figure 7 shows that the position estimation error standard deviation of one INS within the multi-INS fusion without constraints is even lower than that obtained with the single IMU solution. With independent GPS position bias estimations for each INS, the standard deviation would have been approximately the same. The lower standard deviation should be attributed to the common GPS position bias estimation for all the INSs. The estimation of a common bias also has an impact on the velocity (Figure 8) and position (Figure 9) estimations in the final fusion step (average of INS estimations).

However, it should be noted that all INS estimates within the multi-INS solution are corrected with the same GPS and attitude reference sensor measurements and that all INS error states share the same GPS position bias estimation. As the precision of the IMUs decreases, more relative weight is put on the aiding sensor measurements. This, in turn, increases the inter-INS correlation, which may limit the reduction of the estimation error in the final fusion (e.g., if the estimates become 100% correlated among the INSs, their average is the same as each individual INS estimation). Furthermore, the correlation is also increased by the uses of geometrical constraints, especially if the corresponding variances are set with low values or if their update rate is high. However, with the sensor combination and the tuning of the geometrical constraints used in this research, the inter-INS correlation accumulation remains limited so that the impact on the final fusion is negligible.

#### 4.1.2. Test without GPS Receiver

**Figure 10:**
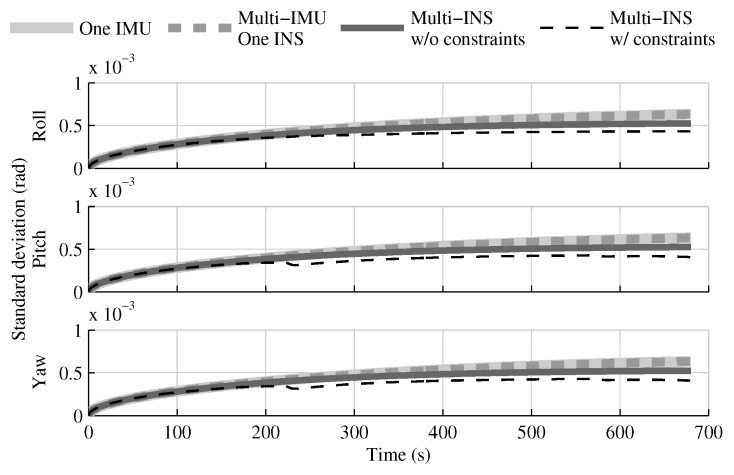
The attitude estimation error standard deviation obtained with the navigation solutions, when the GPS receiver is not present.

**Figure 11:**
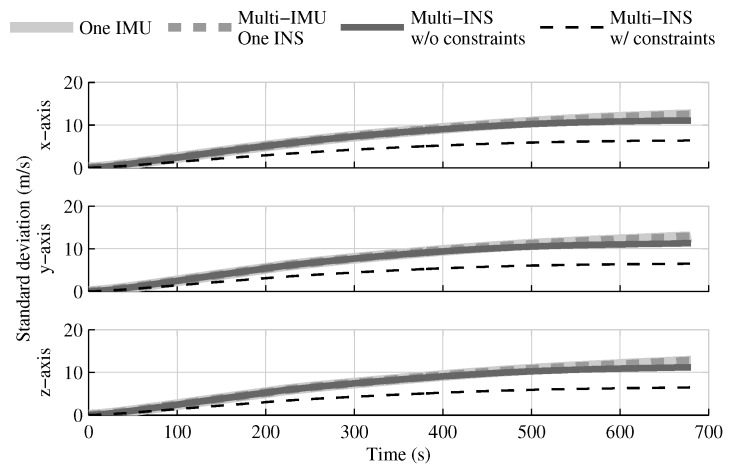
The velocity estimation error standard deviation obtained with the navigation solutions, when the GPS receiver is not present.

**Figure 12:**
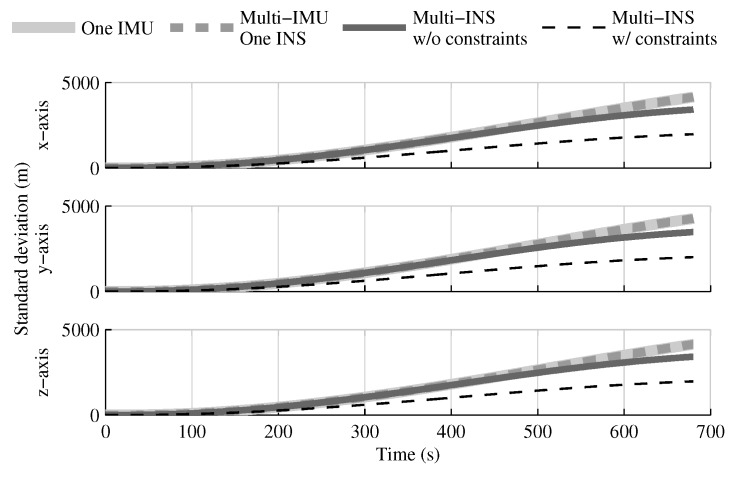
The position estimation error standard deviation obtained with the navigation solutions, when the GPS receiver is not present.

The following tests evaluate the navigation performances without GPS. Again, the solution which fuses all IMUs in one INS generates similar results as the single IMU approach (Figure 10, Figure 11 and Figure 12).

**Figure 13:**
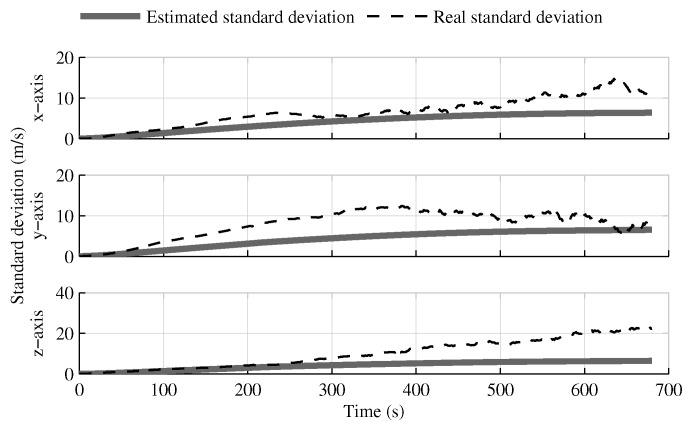
The velocity estimation error standard deviation computed by the navigation filter and the one obtained from Monte Carlo simulation, when the GPS receiver is not present.

**Figure 14:**
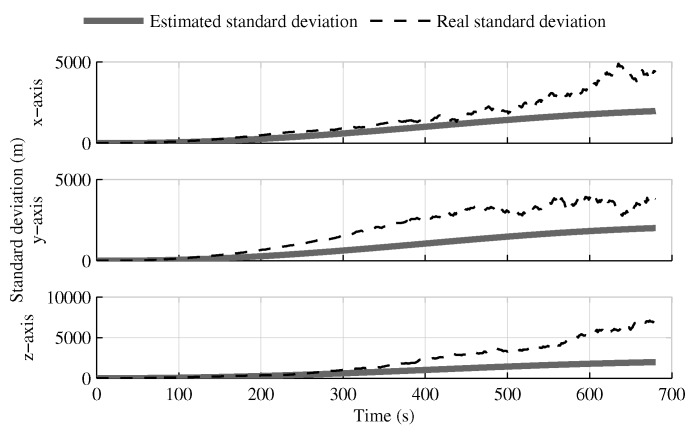
The position estimation error standard deviation computed by the navigation filter and the one obtained from Monte Carlo simulation, when the GPS receiver is not present.

**Figure 15:**
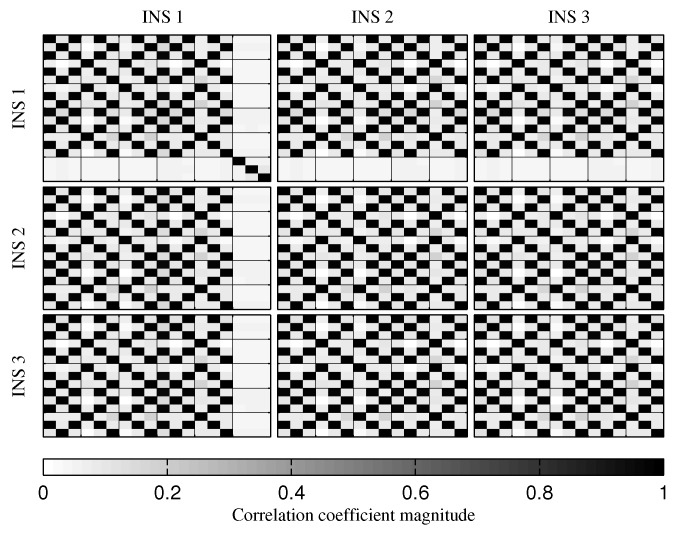
Graphical representation of the correlation between the states of the navigation filter at the 30th second of the simulation, when all geometrical constraints are present.

The fusion of multiple INSs provides better estimates (Figure 10, Figure 11 and Figure 12). The addition of the geometrical constraints theoretically brings a huge improvement on the velocity (Figure 11) and position (Figure 12) estimations. However, the Monte Carlo simulation revealed that the velocity (Figure 13) and position (Figure 14) estimation error standard deviations are underestimated by the navigation filter when the geometrical constraints are present. As stated in the introduction, geometrical constraints provide only relative information and may cause correlation accumulation. Therefore, the velocity and position estimation errors in one INS are distributed among all INSs. The real errors might be different for each INS, but if the estimated errors are equal, the constraints (Equations (22) and (28)) are satisfied. In the absence of absolute measurements, this may lead to filter divergence. Figure 15 shows a graphical representation of the filter estimation correlation matrix when the geometrical constraints are used [43]. White represents uncorrelated states, and black represents 100% correlation, be it negative or positive. The off-diagonal blocks reveal a near perfect correlation between the INS error estimates after only 30 s of simulation. In the absence of GPS measurements, even reducing the update rate of the geometrical constraints has a limited impact on the correlation accumulation.

**Figure 16:**
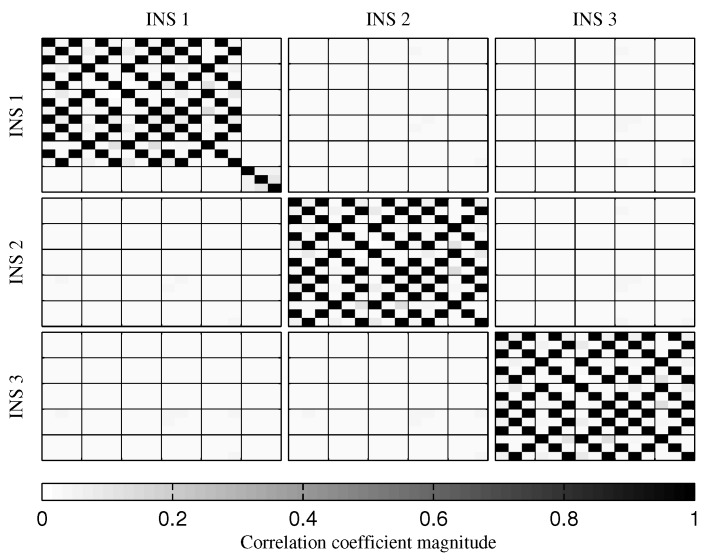
Graphical represetation of the correlation between the states of the navigation filter at the 30th second of the simulation, when only the relative attitude constraint is present.

**Figure 17:**
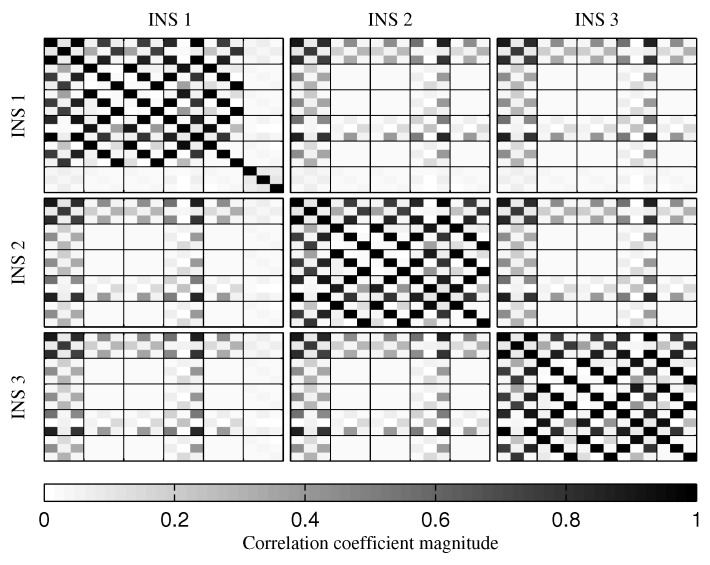
Graphical represetation of the correlation between the states of the navigation filter at the 674th second of simulation, when only the relative attitude constraint is present.

To reduce the correlation accumulation when the GPS receiver is not present, the variance of the relative velocity and position constraints was gradually increased. The results showed that, to prevent excessive correlation accumulation, the variance must be increased to a point where it is equivalent to not use these constraints. Therefore, the use of the relative velocity and position constraints should be avoided during long GPS outages. For comparison, Figure 16 shows the correlation after 30 s of simulation when only the relative attitude constraint is used. As expected, the velocity and position estimation error correlations between the INSs are reduced. The fact though, is that removing the relative velocity and position constraints reduces the inter-INS correlation for all estimated values. There is still correlation accumulation, and, at the end of the mission, the inter-INS correlation for the attitude estimation error remains high (Figure 17). However, the attitude reference sensor measurements provide the attitude observability required to prevent navigation filter divergence [46].

**Figure 18:**
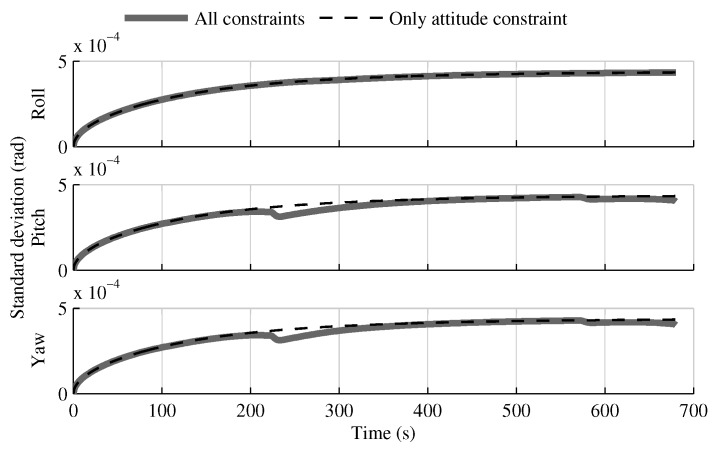
The attitude estimation error standard deviation obtained with the multi-INS fusion when all geometrical constraints are applied versus when only the relative attitude constraint is used.

Removing the relative velocity and position constraints causes the velocity and position estimation error standard deviations to match the solution without constraints. However, the impact on the attitude estimate is minor, as shown in Figure 18.

#### 4.1.3. Conclusion on Sensors Installed in the Launcher Head

The fusion of three IMUs in one INS does not improve the navigation performances, regardless of whether the GPS receiver is present or not. Nonetheless, this approach may be attractive if it results in a cost reduction; however, this analysis is beyond the scope of this research. The fusion of multiple INSs leads to a good reduction in estimation errors, and the addition of geometrical constraints provides even better results. However, when the GPS receiver is not present, the geometrical constraints cause correlation accumulation, which leads the navigation filter to underestimate the velocity and position estimation error standard deviations. Furthermore, excessive inter-IMUs correlation may result in the divergence of the navigation filter. Therefore, during long GPS outages, the relative velocity and position constraints must not be used. Table 2 summarizes the estimation improvements provided by the fusion of multiple INSs.

### 4.2. Tests with IMUs Distributed Along the Launcher Structure

When the IMUs are installed at the top of each stage, sensors are dropped with every jettisoning. However, having sensors distributed along the launcher structure allows for the measurement of the angular dynamics through the accelerometers. Considering the size of a satellite launcher, long lever arms between the IMUs are possible. The objective is to verify the impact of this inertial sensor distribution on the navigation performance.

**Figure 19:**
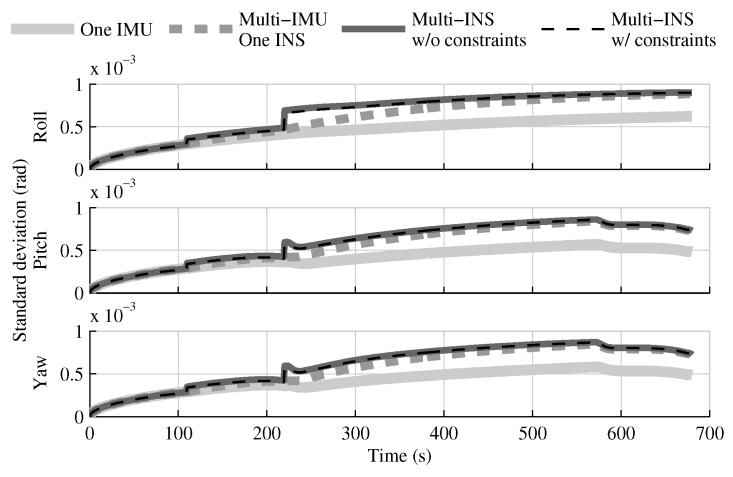
The attitude estimation error standard deviation obtained with the navigation solutions, when the attitude reference sensor and GPS receiver are present.

**Figure 20:**
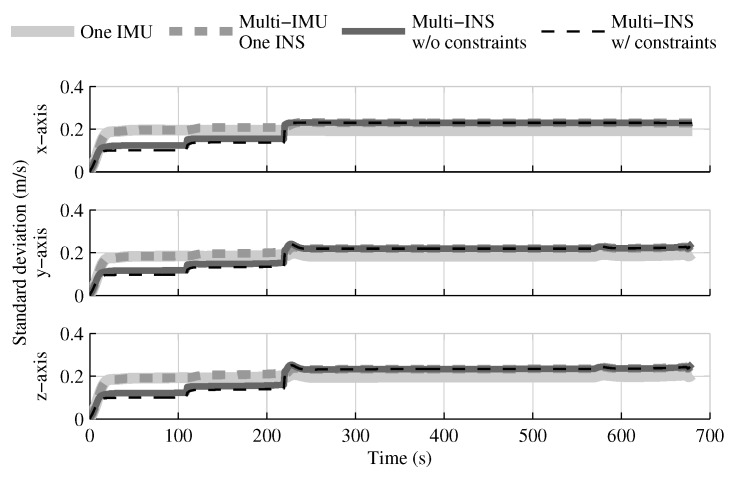
The velocity estimation error standard deviation obtained with the navigation solutions, when the attitude reference sensor and GPS receiver are present.

**Figure 21:**
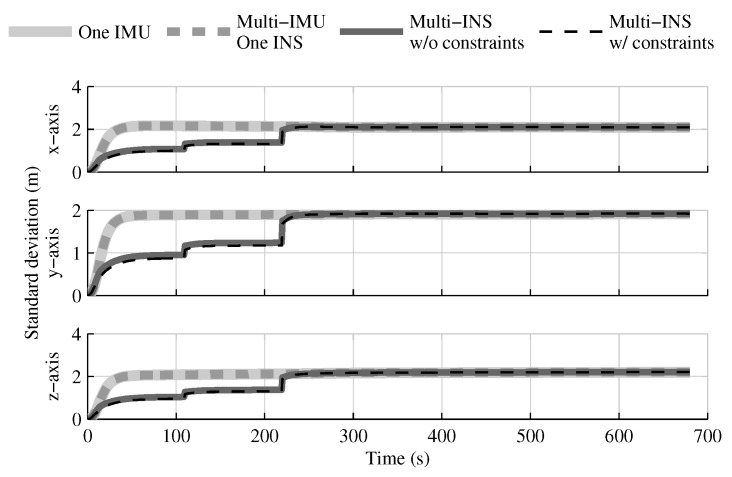
The position estimation error standard deviation obtained with the navigation solutions, when the attitude reference sensor and GPS receiver are present.

Figure 19 shows that, even when all IMUs are present, the attitude estimation is not improved in comparison with the single IMU navigation case, and hence with collocated sensors (Figure 4). Furthermore, once sensors are lost due to the first jettisoning, the estimates of the single IMU solution are better than those of all configurations with distributed sensors. The velocity (Figure 20) and position (Figure 21) estimations are also degraded after the first jettison in comparison to those obtained with collocated sensors (Figure 5 and Figure 6). Nonetheless, even with only one INS, the position estimation error standard deviation of the multi-IMU solutions does not exceed the one obtained with the single IMU solution. The error growth being bounded by the aiding sensor measurements (in this case, the GPS position measurements).

In the fusion of three IMUs in one INS, when sensors are dropped, only their measurements are lost. However, in the fusion of multiple INSs, the data of a complete INS is discarded with each jettison, which explains the quick impact on the estimated standard deviations. To improve the attitude estimates with the IMUs used in this research, the distance between the IMUs must be at least in the order of kilometers. Obviously, a launcher of this size does not exist, as it exceeds the size of current super heavy-lift launch vehicles. Improvements of the attitude and velocity estimates can be obtained when commercial grade gyroscopes are combined with strategic grade accelerometers. Nevertheless, even with this combination of sensors, the improvement is limited to the first boost phase, where all IMUs are present.

**Figure 22:**
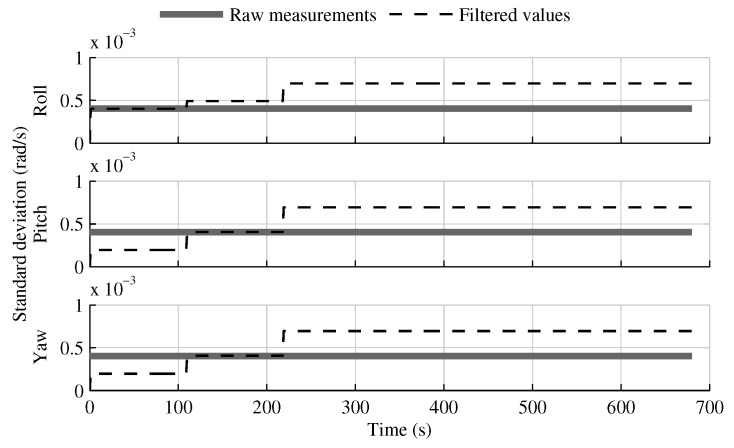
The raw gyroscope measurement standard deviation and the standard deviation of the angular velocity estimated in the fusion of all IMU into one INS, when the attitude reference sensor and GPS receiver are present.

**Figure 23:**
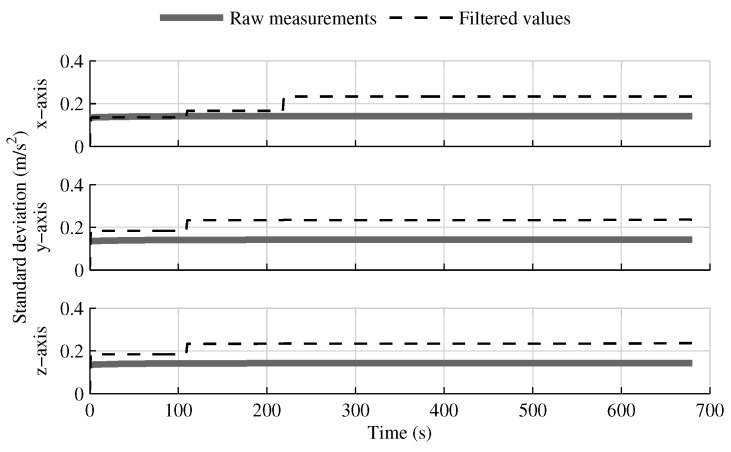
The raw accelerometer measurement standard deviation and the standard deviation of the acceleration estimated in the fusion of all IMU into one INS, when the attitude reference sensor and GPS receiver are present.

The only estimates that benefit from having inertial sensors distributed along the launcher structure are the pitch and yaw angular velocities, which are included in the data fusion model of the solution fusing all IMUs in one INS (Figure 22). The gain is even more evident when the distance between the IMUs increases or when bad gyroscopes are combined with good accelerometers. However, with the sensors used in this research, the gain is limited to the first boost phase. Furthermore, this improvement comes at the cost of worse *y* and *z*-axis acceleration estimations (Figure 23).

When the GPS receiver is not present, the attitude, velocity, and position estimates remain equivalent to those obtained with collocated sensors when all IMUs are present, and are degraded after the first jettisoning. However, the velocity and position standard deviations are no longer underestimated in the fusion of multiple INSs involving all geometrical constraints. This is due to the fact that the geometrical constraints related to the dropped IMUs are removed, which prevents the excessive correlation accumulation effect.

The distribution of the IMUs along the launcher structure is not advantageous with the IMUs selected in this paper. This confirms the fact that the accelerometers generate little information on the angular dynamics, except for the high angular rate applications [29]. Even the long lever arms cannot compensate for the slow angular dynamics of a satellite launcher. Therefore, after the first jettisoning, the attitude, velocity, and position estimation performances are degraded for all navigation solutions when compared to their counterparts involving collocated sensors. Furthermore, with long lever arms, flexible modes can induce measurement disturbances [19], hence reducing the navigation performances obtained here with a rigid launcher and thus the attractiveness of IMU distribution along the launcher structure. However, considering the results obtained when all IMUs are present, if the IMUs are rigidly mounted close to each other in the launcher head, the performances will be equivalent to the one with perfectly collocated sensors. Therefore, the short lever arms, inherent in a real assembly of collocated IMU, are not a concern.

## 5. Conclusions

This paper looks at the effect of navigating a satellite launcher with one IMU versus three IMUs of lower precision and accuracy. Each navigation solution includes a three-axis attitude reference sensor, and all approaches are tested with and without a GPS receiver. First, all sensors are collocated in the launcher head. Then, for the multi-IMU solutions, one IMU is installed at the top of each stage. Three multi-IMU navigation fusion approaches are tested: fusion of all IMUs in one INS, fusion of multiple INSs, and fusion of multiple INSs with geometrical constraints.

The results show that, in a satellite launcher context, the angular velocities are too slow to benefit from distributing the IMUs along the launcher structure. Even with very good accelerometers and bad gyroscopes, or a distance between the IMUs in the order of kilometers, the gains obtained with all IMUs are marginal, and the performances degrade after the first jettisoning. Therefore, installing all sensors in the launcher head leads to the best performances. Fusing all IMUs in one INS does not improve the attitude, velocity, and position estimates in comparison to the single IMU solution. However, the fusion of multiple INSs provides good improvement, and the addition of geometrical constraints further reduces the estimation errors. During long GPS outages, the use of the relative velocity and position constraints must be avoided in order to prevent excessive correlation accumulation.

This research opens many opportunities for future works:

- evaluating the impact of using more than three IMUs in the fusion of multiple INSs, either with or without geometrical constraints;
- testing the fusion of multiple INSs with geometrical constraints, when independent GPS receivers and attitude reference sensors are used for each INS;
- investigating the reasons which lead the Kalman filter to diverge when the GPS receiver and attitude reference sensor measurement correlations among INSs are considered in the observation covariance matrix;
- comparing the impact of including the knowledge of the launcher dynamics in the fusion of multiple IMUs in one INS to the one obtained by the authors in a previous work with a single IMU.

## Figures and Tables

**Table 1:** Sensor specifications.

GPS Receiver	C/A Code with Wide Correlator
Attitude reference sensor noise standard deviation	$1^{{}^{\circ}}$
Gyroscope random walk (multi-IMUs)	$0.72^{{}^{\circ}}/h/\sqrt{Hz}$
Gyroscope bias stability (multi-IMUs)	$0.05^{{}^{\circ}}/h$
Accelerometer random walk (multi-IMUs)	117 μg/$\sqrt{Hz}$
Accelerometer bias stability (multi-IMUs)	7500 μg
Gyroscope random walk (single-IMU)	$0.416^{{}^{\circ}}/h/\sqrt{Hz}$
Gyroscope bias stability (single-IMU)	$0.029^{{}^{\circ}}/h$
Accelerometer random walk (single-IMU)	68 μg/$\sqrt{Hz}$
Accelerometer bias stability (single-IMU)	4330 μg

**Table 2:** Reduction of the estimation error standard deviations provided by the fusion of multiple INSs at the satellite injection (values in parentheses represent the maximum improvement when it does not occur at the satellite injection).

		With GPS Receiver		Without GPS Receiver	
		w/o Constraint	All Constraints	w/o Constraint	Attitude Constraint
Attitude	roll	16%	31%	17%	32%
	pitch	16%	28%	17%	32%
	yaw	17%	29%	17%	32%
Velocity	x	39%	51%	11%	11%
	y	35% (40%)	48% (52%)	12%	12%
	z	35% (41%)	49% (52%)	11%	12%
Position	x	50% (54%)	54% (59%)	18%	18%
	y	50% (54%)	53% (59%)	19%	19%
	z	50% (54%)	54% (59%)	18%	18%
